# Sacituzumab govitecan in metastatic breast cancer: a systematic review and meta-analysis of its clinical benefit and safety

**DOI:** 10.3389/fmed.2026.1948167

**Published:** 2026-09-18

**Authors:** Zepeng Wang, Shewen Lyu, Yujing Mu, Xiaohua Pei

**Affiliations:** Department of Breast Surgery, Beijing University of Chinese Medicine Third Affiliated Hospital, Beijing, China

**Keywords:** adverse events, antibody-drug conjugate, breast cancer, clinical therapeutic effect, sacituzumab govitecan, systematic review

## Abstract

**Introduction:**

Trophoblast cell surface antigen 2 (Trop-2), a transmembrane glycoprotein abundantly expressed in breast cancer tissues, has become a prominent target in precision cancer therapy, with Sacituzumab govitecan (SG) being the first antibody-drug conjugate (ADC) designed against it. Breast cancer, the most common malignancy in women, has a prognosis severely compromised by recurrence and metastasis. This meta-analysis of randomized controlled trials (RCTs) aims to systematically evaluate the clinical benefits and safety of SG in treating metastatic breast cancer (mBC). (https://www.crd.york.ac.uk/PROSPERO/view/CRD420261347978).

**Methods:**

A systematic search was carried out in PubMed, Embase, the Cochrane Library, and ClinicalTrials.gov, with a search period extending up to December 1, 2025, for RCTs examining the therapeutic efficacy and safety of SG for mBC. All statistical analyses were conducted using Review Manager (version 5.4), with pooled effect sizes computed using a fixed-effect or random-effects model depending on the degree of heterogeneity.

**Results:**

A total of four phase III, multicenter, open-label RCTs were included in this systematic review. The experimental arm received sacituzumab govitecan (SG), and the control arm received chemotherapy. The efficacy analysis included 1,900 patients with metastatic breast cancer (mBC) (SG arm, *n* = 952; control arm, *n* = 948), and the safety analysis included 1,879 patients (SG arm, *n* = 966; control arm, *n* = 913). Compared with chemotherapy, SG significantly prolonged progression-free survival (PFS) (HR 0.58, 95% CI 0.46–0.72) and overall survival (OS) (HR 0.62, 95% CI 0.45–0.86). In addition, SG showed a trend toward higher objective response rate (ORR) and partial response (PR) rate, though neither difference was statistically significant; however, the complete response (CR) rate was significantly higher with SG (RR 1.94, 95% CI 1.11–3.38). The any-grade adverse events (AEs) with the highest reported incidence were neutropenia, followed by nausea, diarrhea, alopecia, anemia, and fatigue. Notably, treatment with SG was linked to a significantly elevated rate of grade ≥3 neutropenia (RR 1.24, 95% CI 1.06–1.46) and diarrhea (RR 8.46, 95% CI 1.48–48.51).

**Conclusion:**

SG may provide clinical benefit and favorable effects on HRQoL in mBC; however, its more definitive clinical advantages and the magnitude of benefit still require further validation. In addition, SG is associated with toxicities including neutropenia and diarrhea, warranting appropriate prophylactic measures in clinical practice.

**Systematic Review Registration:**

https://www.crd.york.ac.uk/PROSPERO/view/CRD420261347978, PROSPERO CRD420261347978.

## 1 Introduction

According to the latest global cancer statistics published by the International Agency for Research on Cancer (IARC), there were approximately 2.3 million newly diagnosed cases of female breast cancer worldwide in 2022, making it the second most frequently diagnosed malignancy overall. It remains the most common invasive cancer among women and the leading cause of cancer-related mortality in this population (1). Breast cancer is classified into distinct molecular subtypes based on the immunohistochemical expression of estrogen receptor (ER), progesterone receptor (PR), and human epidermal growth factor receptor 2 (HER2) in tumor tissue. The principal clinical subtypes are triple-negative breast cancer (TNBC), hormone receptor-positive/HER2-negative (HR+/HER2−) disease, and HER2-positive breast cancer (2). Therapeutic selection is subtype-specific and further guided by biological features such as proliferative activity; standard modalities include cytotoxic chemotherapy, endocrine therapy, HER2-targeted agents, and immune checkpoint inhibitors (3).

Previous studies have shown that at primary diagnosis, 3%–8% of breast cancer patients present with metastatic disease, whereas 30%–40% of those with early-stage disease ultimately develop distant recurrence (4, 5). In metastatic breast cancer (mBC), the relative 5-year survival rate is merely 31% (6). Despite recent advances in chemotherapy, targeted therapy, endocrine therapy, and immunotherapy, the management of mBC remains challenging because of tumor cell drug resistance, limited therapeutic targets, and potentially substantial toxicity associated with combination regimens. These factors collectively limit survival benefits in patients with mBC.

Antibody-drug conjugates (ADCs) consist of cytotoxic drugs (i.e., drug payloads) linked to monoclonal antibodies that target tumor-specific antigens via a linker (7). This enables precise targeting to the tumor site, allowing for efficient killing of cancer cells while minimizing damage to normal tissues (8). The specific combination of components determines the therapeutic efficacy and safety profile of ADCs. ADCs represent a landmark technological achievement in tumor-targeted therapy over the past decade, offering highly promising treatment strategies for various types of cancer. Among validated ADC targets, trophoblast cell surface antigen 2 (Trop-2), a transmembrane glycoprotein encoded by the *TACSTD2* gene, displays minimal expression in most normal tissues but is consistently overexpressed across multiple epithelial malignancies, including breast, urothelial, and lung cancers (9). Trop-2 promotes the proliferation, invasion, and metastatic dissemination of tumor cells, and has thus emerged as a significant target garnering extensive attention in the era of precision oncology (10).

Sacituzumab govitecan (SG), the pioneering anti-Trop-2 ADC globally, initially received accelerated approval from the U.S. Food and Drug Administration (FDA) in April 2020 for the treatment of adult patients with metastatic TNBC who had received at least two prior therapies for metastatic disease (11), and was granted full approval in April 2021. SG is synthesized by chemically conjugating the Trop-2-targeting monoclonal antibody hRS7 IgG1*κ* to the payload SN-38, the active metabolite of irinotecan, via the CL2A linker (12). Upon systemic delivery, SG selectively recognizes and binds to Trop-2 receptors overexpressed on tumor cell membranes. Following endocytosis, the CL2A linker is cleaved to release SN-38, which subsequently inhibits DNA and RNA synthesis in tumor cells and exerts antitumor cytotoxicity. Although SN-38 is a moderately cytotoxic agent, the high drug-to-antibody ratio (DAR) of 7.6 and the moderately stable linker of SG effectively enhance its overall antitumor activity (13). The IMMU-132-01 trial, a first-in-human, single-arm, basket study, assessed the safety and efficacy of SG across advanced solid malignancies (14). In patients with metastatic TNBC who had received prior therapy, SG conferred substantial clinical benefit, as evidenced by prolonged response duration, extended progression-free survival (PFS), and improved overall survival (OS) (15).

In recent years, clinical trials evaluating SG in the treatment of metastatic solid tumors have been continuously conducted, and accumulating clinical evidence indicates that SG confers long-term survival benefits for patients with mBC (16). Although several meta-analyses of SG have been published, most existing studies have focused on broad solid tumor populations or have primarily addressed safety events (17); a comprehensive integrated analysis of survival data and safety profiles across all subtypes of mBC remains lacking. Therefore, this study aims to systematically evaluate the benefits and adverse event profiles of SG in patients with mBC by including high-quality randomized controlled trials (RCTs) with comparable inclusion criteria and intervention protocols, and by pooling a larger sample size and the most recent data (including updated results from the ASCENT-03 trial), thereby providing evidence to inform the optimal clinical application of this therapy.

## 2 Materials and methods

### 2.1 Search strategy and registration

A systematic search for RCTs was carried out in PubMed, Embase, the Cochrane Library, and the U.S. Clinical Trials Registry (ClinicalTrials.gov), with a search period extending up to December 1, 2025. The search strategy included terms for “breast cancer” and “sacituzumab govitecan”, combined with appropriate Boolean operators and MeSH terms as applicable (The complete search strategy is provided in the Appendix). Following the Preferred Reporting Items for Systematic Reviews and Meta-Analyses (PRISMA) Statement (18), the study protocol was prospectively registered on the International Prospective Register of Systematic Reviews (PROSPERO; CRD420261347978) to ensure transparency and traceability.

### 2.2 Eligibility criteria

Inclusion criteria: (1) standard RCTs; (2) patients with histopathologically and radiologically confirmed metastatic or advanced breast cancer (irrespective of tumor molecular subtype); (3) RCTs evaluating SG versus chemotherapy: subjects in the intervention arm were administered SG, whereas those in the control arm received chemotherapy; (4) outcome measures including complete and available survival endpoints (e.g., PFS) and safety profiles (incidence of drug-related adverse events). Exclusion criteria: (1) non-RCT studies; (2) duplicate publications of the same study (with the identical clinical trial registration number); (3) studies whose full texts could not be retrieved (including the meeting summary); and (4) studies that provided no data on the prespecified outcomes.

### 2.3 Data extraction and quality assessment

Two reviewers independently performed the tasks of literature screening and data extraction, collecting baseline information from each eligible study, comprising: (1) study title and clinical trial registration number; (2) study center(s); (3) study period and publication year; (4) first author; (5) trial phase and design type; and (6) specific details of the experimental and control groups, including sample size, age, gender, breast cancer subtype, and specific interventions. Outcome data were also extracted. Risk of bias appraisal was undertaken, with any disagreements resolved via mutual consultation or adjudication by an independent third reviewer. The Cochrane RoB 2 tool was employed to evaluate the methodological quality of the included RCTs by inspecting five potential sources of bias; subsequently, studies were classified as low risk, high risk, or some concerns (19).

### 2.4 Outcome measures

The primary outcome measures were survival-related endpoints, specifically PFS evaluated through blinded independent central review (BICR) and overall survival (OS). Key secondary measures consisted of the objective response rate (ORR, defined as the sum of complete and partial responses), the frequency of any-grade common adverse events (AEs) including neutropenia, diarrhea, anemia, alopecia, and fatigue, as well as the incidence of grade ≥3 AEs.

### 2.5 Statistical analysis

All statistical analyses were conducted using Review Manager (version 5.4). Heterogeneity across studies was assessed through the *I^2^* statistic and the Cochrane *Q* test. In cases where substantial heterogeneity was present (*I^2^* ≥ 50%, *Q* test *P* < 0.1), effect sizes were pooled using a random-effects model based on the DerSimonian–Laird method; otherwise, a fixed-effect model (inverse-variance or Mantel-Haenszel approach) was adopted (20). Survival outcomes were expressed as hazard ratios (HR) with corresponding 95% confidence intervals (CIs), whereas dichotomous endpoints were assessed using risk ratios (RR) with their 95% CIs. We defined statistical significance as *P* < 0.05.

The following approaches were employed to explore heterogeneity in this study: (1) Sensitivity analysis was performed using a leave-one-out approach, wherein each study was systematically removed one at a time to assess its individual impact on the overall pooled effect size. (2) Where sufficient data permitted, pre-specified exploratory subgroup analyses (e.g., by tumor type) were performed.

## 3 Results

### 3.1 Study eligibility assessment

Through systematic retrieval of the PubMed, Embase, Cochrane Library and ClinicalTrials.gov databases, a total of 456 records were obtained and included in the preliminary screening phase. After the removal of duplicate records across databases (*n* = 224), 232 articles remained for initial assessment. Of the records screened by title and abstract, 172 were excluded for failing to meet the inclusion criteria, with reasons including being a review, systematic review, or commentary, having irrelevant study content, or employing incompatible interventions or comparators. A total of 60 articles underwent full-text evaluation, from which four trials fulfilled the eligibility requirements. These four RCTs were subsequently incorporated into the quantitative synthesis. Figure 1 presents the PRISMA flow diagram summarizing the process of literature screening and selection.

**Figure 1:**
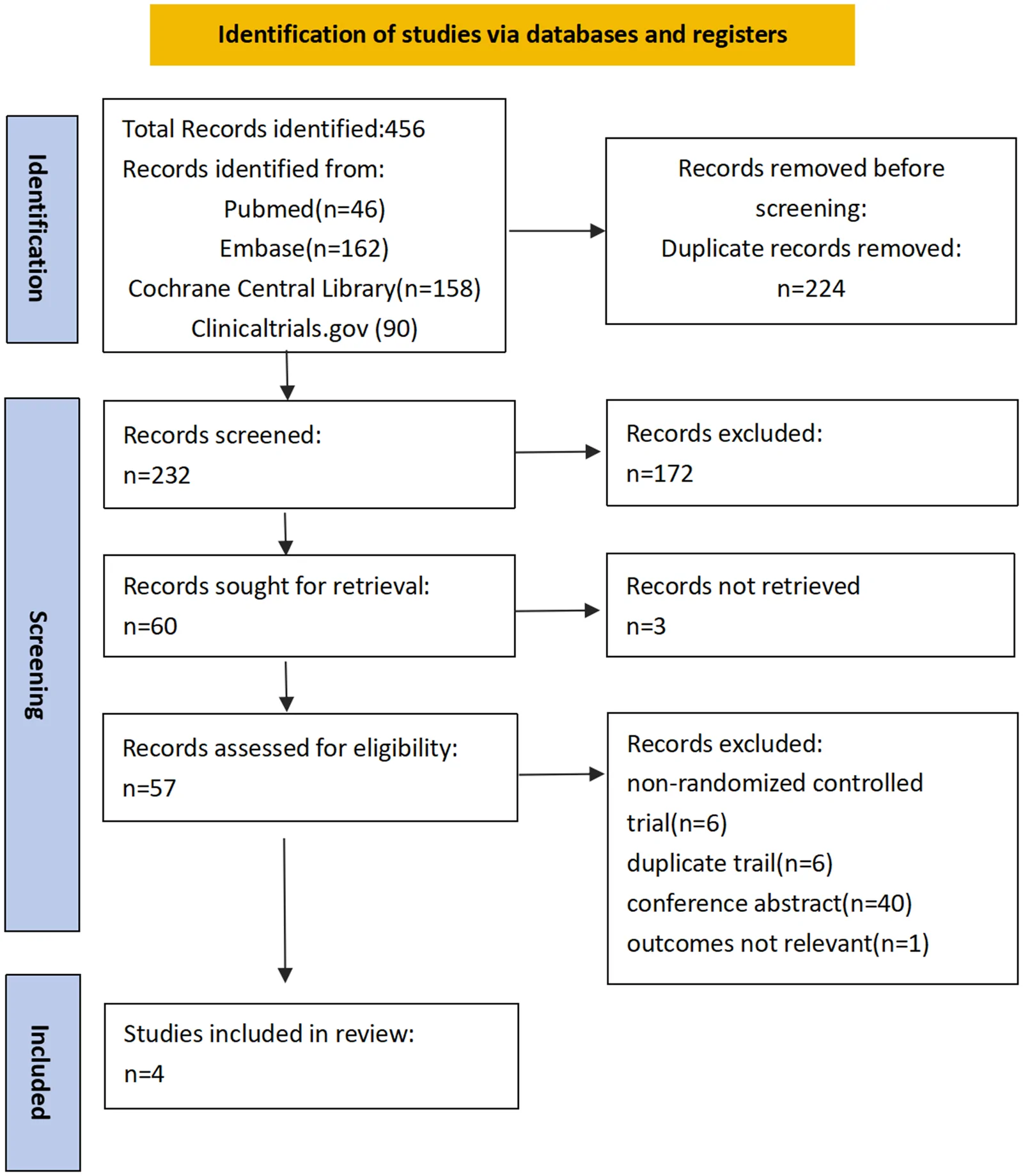
Study selection flowchart according to PRISMA guidelines.

Study quality and risk of bias were critically appraised in order to safeguard the validity of the meta-analytic results, before proceeding to data synthesis. The four included studies (21–24) were all open-label RCTs; however, assessment of the primary outcomes was performed by a BICR. Consequently, the RoB 2 tool indicated that all four trials had a low risk of bias; no issues were found in either the randomization process or outcome measurement. The outcomes of the bias evaluation are displayed in Figure 2.

**Figure 2:**
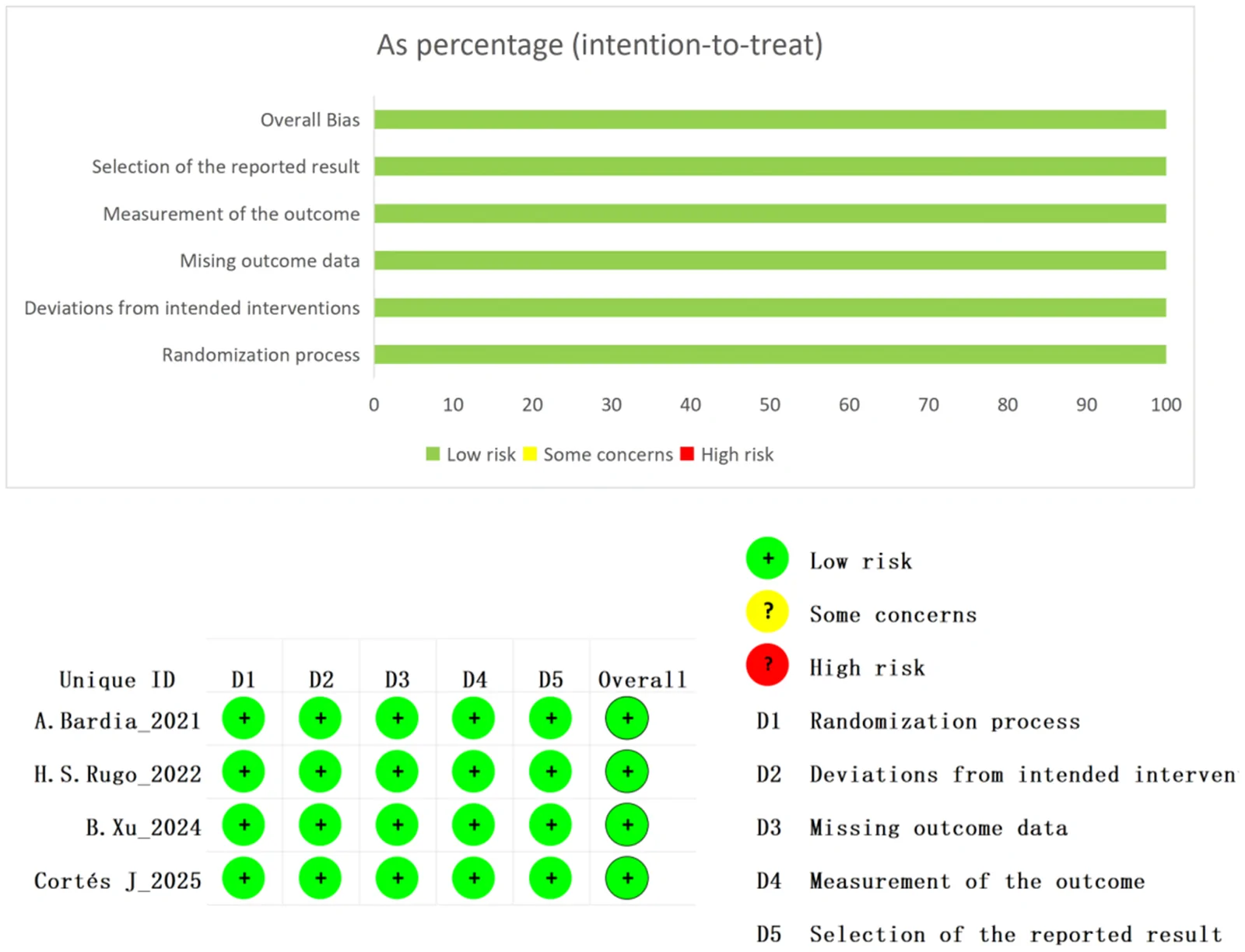
Risk of bias.

### 3.2 Baseline characteristics

A total of four studies were included in this systematic review, all of which were phase III, multicenter, open-label RCTs. Two RCTs (21, 24) were conducted in patients with advanced/metastatic TNBC, while the remaining two RCTs (22, 23) evaluated HR+/HER2- breast cancer. The experimental arm received treatment with SG, while the control arm received physician-selected chemotherapy regimens, including taxanes, eribulin, vinorelbine, and capecitabine. BICR-assessed PFS served as the primary endpoint, and secondary endpoints encompassed OS, ORR, duration of response (DOR), and safety assessments.

Specifically, the ASCENT trial (21) enrolled 529 patients with mBC who underwent randomization; among them, 61 patients had brain metastases at baseline, and the remaining 468 patients without baseline brain metastases constituted the primary efficacy analysis population (235 in the SG arm and 233 in the control arm). The safety analysis population included all patients who received at least one dose of study treatment, regardless of the presence of brain metastases at baseline (258 in the SG arm and 224 in the control arm). In the TROPiCS-02 trial (22), the SG and control arms comprised 272 and 271 patients, respectively; in the EVER-132-002 trial (23), 166 and 164 patients; and in the ASCENT-03 trial (24), 279 and 279 patients. In these three trials, efficacy analyses included all randomized patients (intention-to-treat population), and all patients who received at least one dose of study drug were included in the safety analysis.

Therefore, the efficacy assessment in this study encompassed a total of 1,900 patients with mBC (SG arm *n* = 952, control arm *n* = 948), and the safety assessment included 1,879 patients (SG arm *n* = 966, control arm *n* = 913). Due to the immaturity of the OS data at the analysis cutoff, one study (24) was omitted from the pooled OS analysis. The safety assessment was conducted by classifying AEs based on the National Cancer Institute Common Terminology Criteria for Adverse Events (NCI-CTCAE) grading system, with three studies (22–24) employing version 5.0 and one study (21) employing version 4.03. Table 1 presents a comprehensive overview of baseline characteristics and pertinent study data.

**Table 1:** Characteristics at baseline for the eligible studies.

Study/ID	Authors/year	Study type	Location	Paricipant population	Male/famale(n)	Treatment regimen	Median age	Outcomes
ASCENT/ NCT02574455	A.Bardia,et al/ 2021	phase III RCT	88 sites in seven countries	mTNBC; prior exposure to two or more standard chemotherapy regimens for advanced or metastatic disease	2/466	**SG(*n*** **=** **235):** A 21-day cycle with 10 mg/kg i.v. administered on days 1 and 8. **Control(*n*** **=** **233):** Eribulin(54%), vinorelbine(20%), capecitabine(13%), and gemcitabine(12%)	**SG** 54 (29–82) **Control:** 53 (27–81)	PFS; OS, OR, safety.
TROPiCS-02/ NCT03901339	H.S.Rugo,et al/ 2022	phase III RCT	91 sites across North America and Europe	HR+/HER2- mBC; 2–4 prior lines of palliative systemic chemotherapy	5/538	**SG(*n*** **=** **272):** A 21-day cycle with 10 mg/kg i.v. administered on days 1 and 8. **Control(*n*** **=** **271):** Eribulin(48%), vinorelbine(23%), gemcitabine(21%), and capecitabine(8%)	**SG:** 57 (29–86) **Control:** 55 (27–78)	PFS; OS, OR,CBR, DOR, patient-reported outcomes, safety.
Study/ID	Authors/ Year	Study Type	Location	Paricipant Population	Male/ Famale(n)	Treatment regimen	Median Age	Outcomes
EVER-132-002/NCT04639986	B.Xu, et al/ 2024	phase III RCT	41 participating sites spanning China, Taiwan, and the Republic of Korea	HR+/HER2- mBC; endocrine-resistant, previously treated	2/329	**SG(*n*** **=** **166):** A 21-day cycle with 10 mg/kg i.v. administered on days 1 and 8. **Control(*n*** **=** **165):** Eribulin(79%), vinorelbine(8%), capecitabine(7%), gemcitabine(6%)	**SG:** 53 (32–72) **Control:** 51 (28–79)	PFS; OS, OR,CBR,DoR, patient-reported outcomes, safety.
ASCENT-03/NCT05382299	Cortés J,et al/2025	phase III RCT	229 sites in 30 countries	mTNBC; had no history of systemic treatment for advanced disease and were ineligible for PD-1/PD-L1 inhibitor therapy	3/555	**SG(*n*** **=** **279):** A 21-day cycle with 10 mg/kg i.v. administered on days 1 and 8. **Control(*n*** **=** **279):** Taxane(56%), gemcitabine plus carboplatin(44%)	**SG:** 56 (28–84) **Control:** 54 (23–86)	PFS; OS,OR,DoR, patient-reported outcomes,safety.

### 3.3 Survival outcomes

#### 3.3.1 Progression-free survival

When the four studies were pooled, the heterogeneity test *I^2^* = 72% and Cochran's *Q*-test *P* = 0.01, indicating the presence of heterogeneity among the included studies; therefore, a random-effects model was selected for the meta-analysis of effect sizes. The results showed that compared with chemotherapy, SG treatment significantly prolonged PFS in patients with mBC (HR 0.58; 95% CI 0.46–0.72; *Z* = 4.83; *P* < 0.001) (Figure 3). This indicated that patients receiving SG had a 42% reduction in the risk of disease progression.

**Figure 3:**
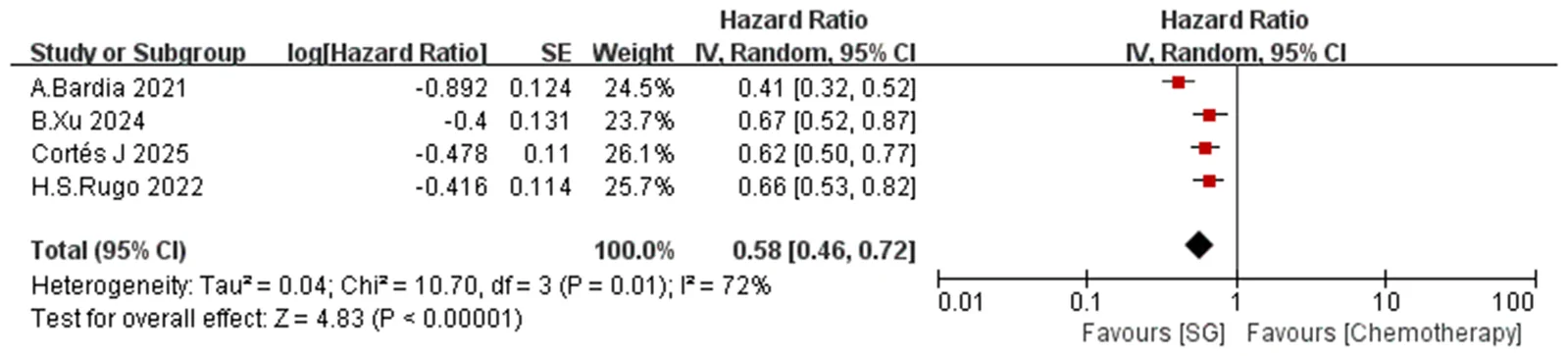
Forest plot of PFS for mBC.

Sensitivity analysis using the leave-one-out approach was performed to explore the source of heterogeneity. After excluding the ASCENT trial, no heterogeneity was observed among the remaining three studies (*I^2^* = 0%, Cochran's Q-test = 0.88). The pooled effect size under the random-effects model was HR = 0.65 (95% CI 0.57–0.74; Z = 6.42, *P* < 0.001) (Figure 4A), and the fixed-effect model yielded a consistent result (HR 0.65; 95% CI 0.57–0.74; Z = 6.42, *P* < 0.001) (Supplementary Figure 1). Considering the clinical context in which these trials were conducted, this discrepancy may be attributable to the fact that the ASCENT trial enrolled heavily pretreated patients with metastatic TNBC, a tumor subtype characterized by high aggressiveness and strong drug resistance, with poor sensitivity to chemotherapy; these patients may therefore derive greater relative benefit from SG compared with chemotherapy, thereby yielding a more favorable HR in the SG arm. Differences in prior treatment history may account for the heterogeneity (Figure 4).

**Figure 4:**
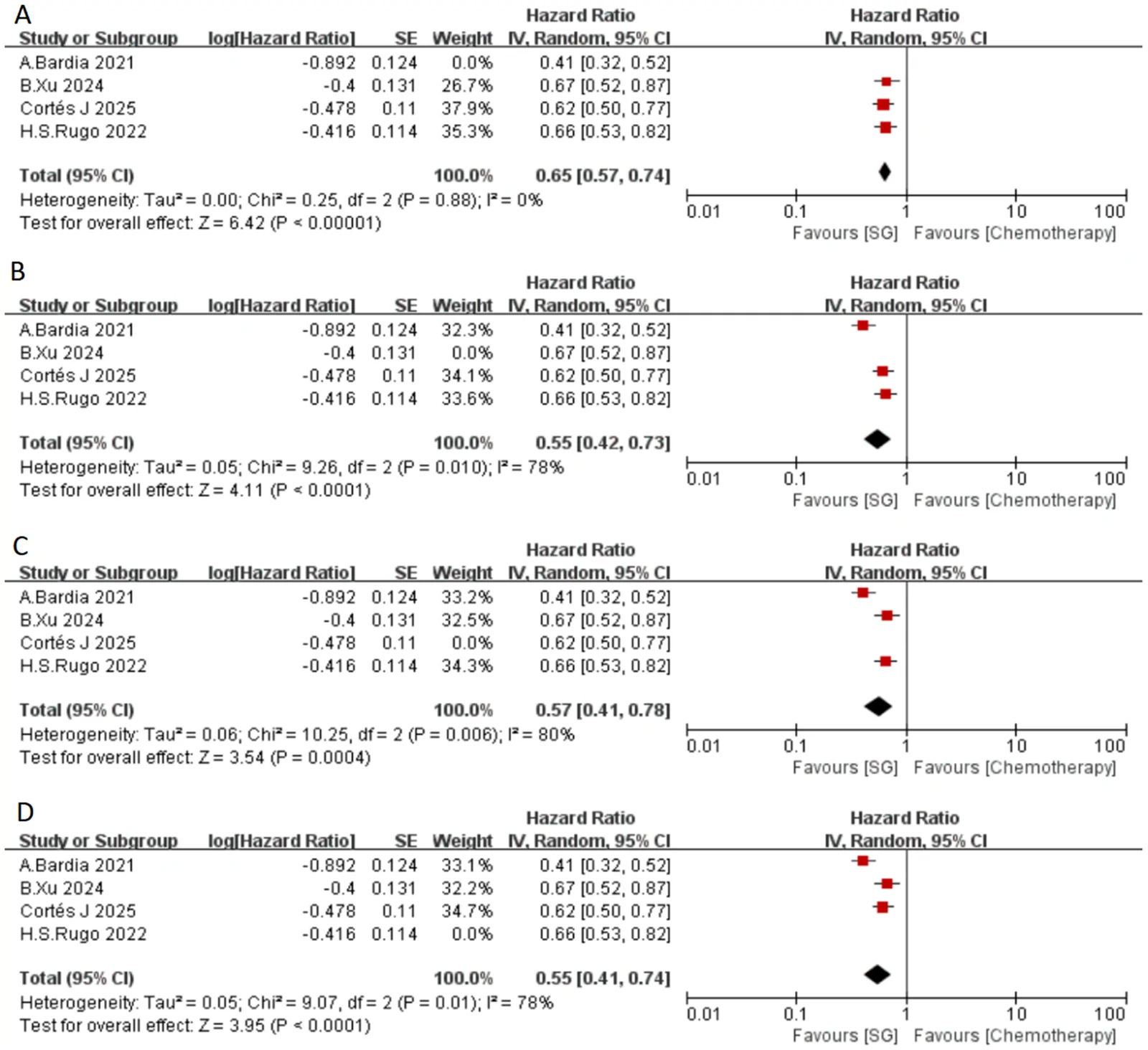
Forest plot for the sensitivity analysis of PFS.

Exploratory subgroup analysis by tumor type showed clinical benefit with SG across all subtypes. In metastatic TNBC, SG reduced the risk of disease progression by 49% versus chemotherapy (HR 0.51; 95% CI 0.34–0.76; *P* = 0.001); in HR+/HER2− disease, the reduction was 34% (HR 0.66; 95% CI 0.56–0.79; *P* < 0.001). However, no statistically significant difference in treatment effect was observed between the two subgroups (*P* = 0.22) (Figure 5). There was substantial heterogeneity between the ASCENT and ASCENT-03 trials (*I^2^* = 84%, *Q*-test *P* = 0.01), likely due to differences in prior treatment: The former enrolled patients who had received at least two prior lines of therapy, whereas the latter included those who had received no prior systemic therapy for advanced disease.

**Figure 5:**
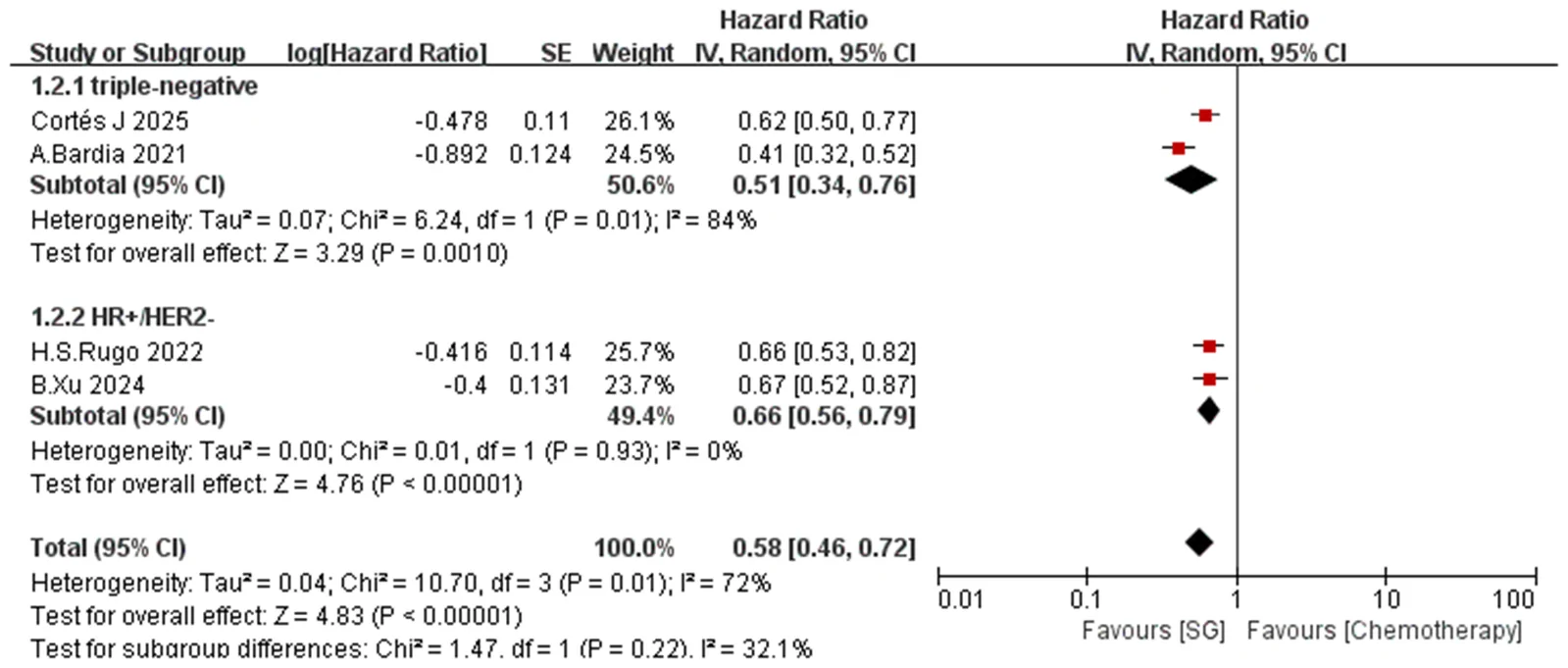
Subgroup analysis of PFS by tumor subtype.

#### 3.3.2 Overall survival

Three studies reported mature OS data. Pooled analysis of OS data from the three studies revealed significant heterogeneity (*I^2^* = 82%, *Q*-test *P* = 0.004). Using a random-effects model, SG significantly prolonged OS compared with chemotherapy in patients with mBC, corresponding to a 38% reduction in the risk of death (HR 0.62, 95% CI 0.45–0.86, *Z* = 2.86, *P* = 0.004) (Figure 6).

**Figure 6:**
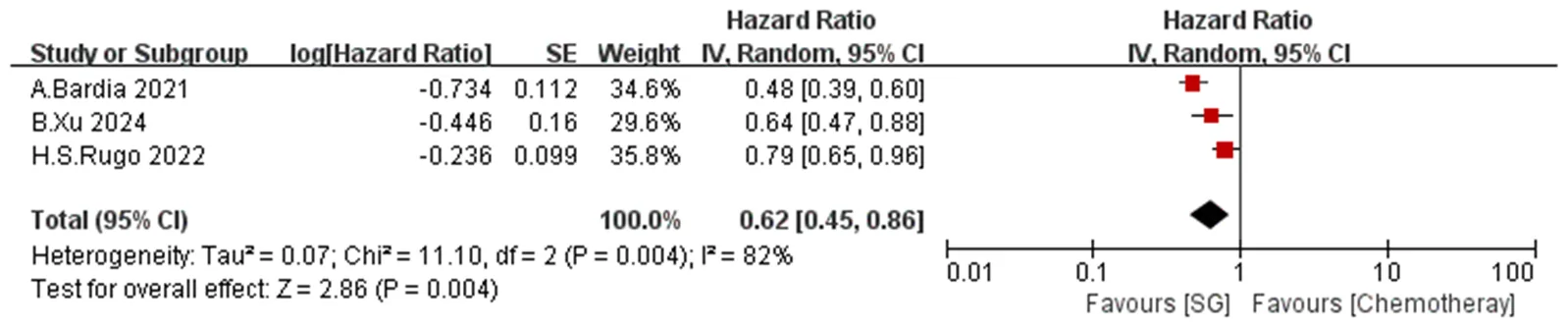
Forest plot of OS for mBC.

To assess the robustness of the results and explore the source of heterogeneity, a leave-one-out sensitivity analysis was further performed. Consistent with the previous section, when the ASCENT trial was excluded, heterogeneity decreased substantially, yielding a pooled HR of 0.74 (95% CI 0.61–0.89, *P* = 0.002) (Figure 7A). Notably, when the EVER-132-002 trial was excluded, the pooled effect size was HR 0.62 (95% CI 0.38–1.01, *P* = 0.05), bringing the pooled estimate to the threshold of statistical significance (Figure 7B).

**Figure 7:**
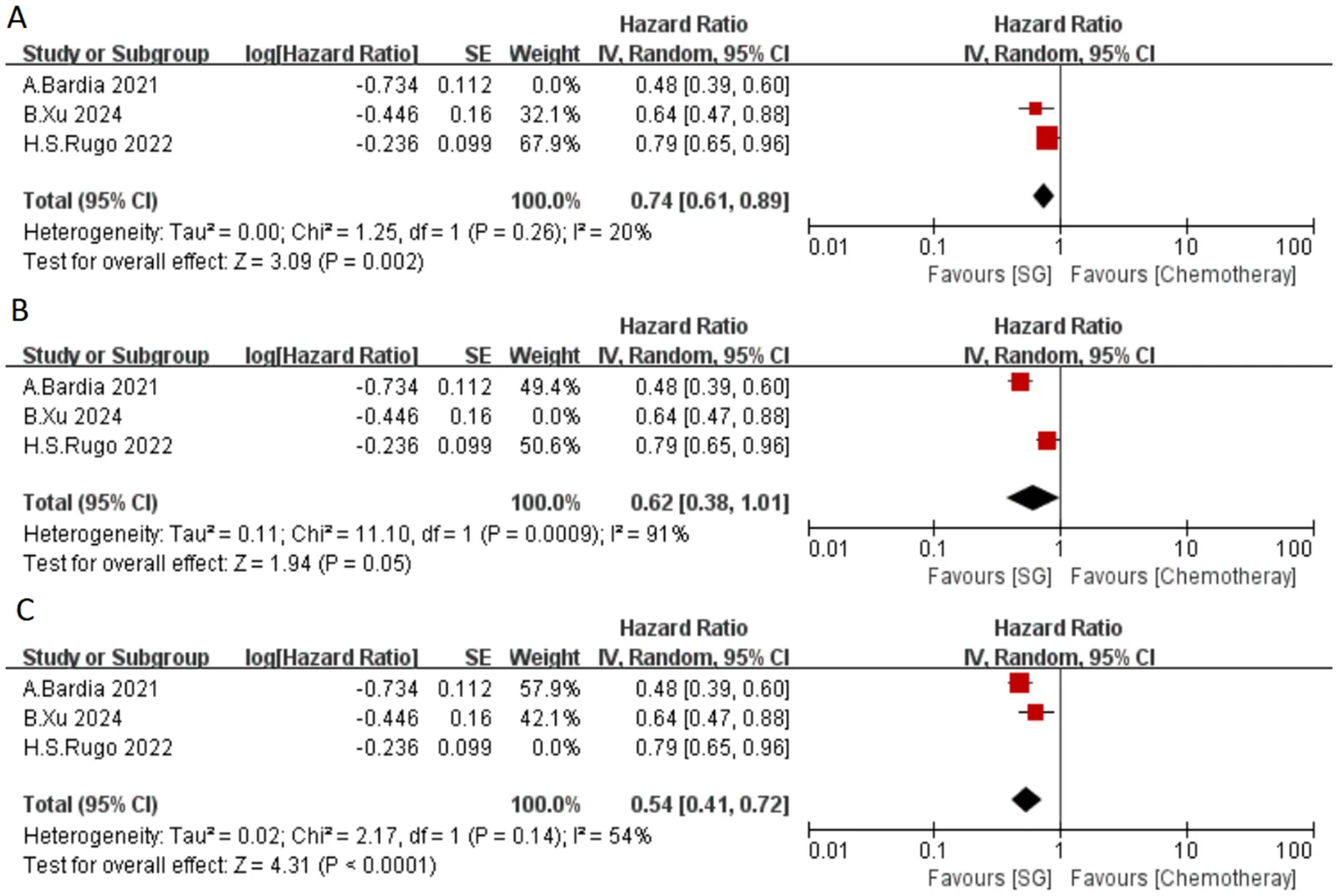
Forest plot for the sensitivity analysis of OS.

### 3.4 Tumor response outcomes

The ORR to tumor therapy consists of complete response (CR) and partial response (PR). Pooled analysis of ORR across the four studies revealed significant heterogeneity (*I^2^* = 93%, *Q*-test *P* < 0.01); hence, a random-effects model was applied. The ORR was higher with SG than with chemotherapy, but not significantly (RR = 1.92, 95% CI 0.96–3.84, *P* = 0.07). No significant heterogeneity was observed for CR (*I^2^* = 15%, *Q*-test *P* = 0.32), and a fixed-effect model showed a significant advantage of SG over chemotherapy (RR 1.94, 95% CI 1.11–3.38; *P* = 0.02). The PR rate, analyzed with a random-effects model due to significant heterogeneity (*I^2^* = 92%, *Q*-test *P* < 0.01), was also higher with SG but not significantly (RR 1.83, 95% CI 0.92–3.64; *P* = 0.08) (Figure 8).

**Figure 8:**
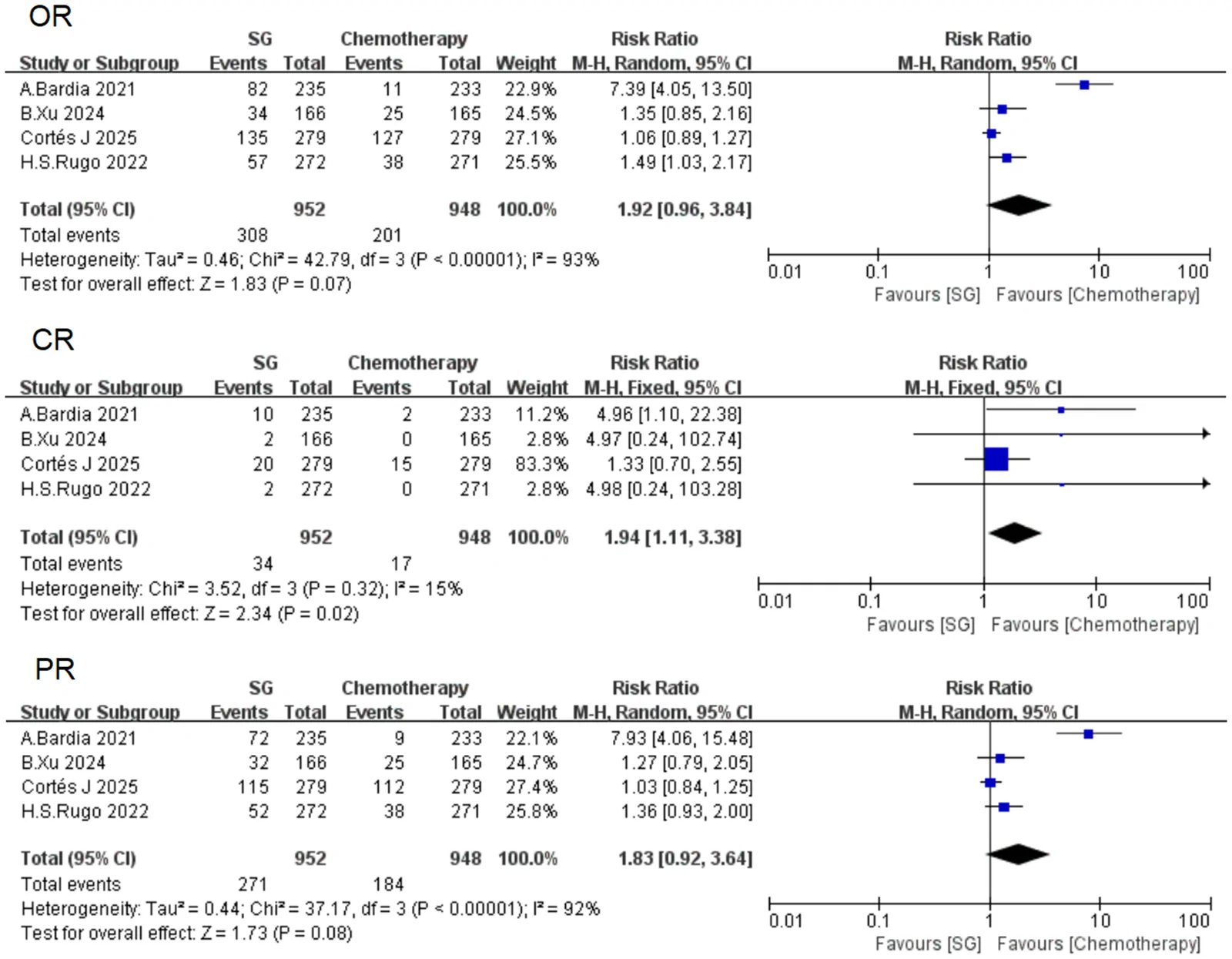
Forest plot of tumor response outcomes for mBC.

Sensitivity analysis of ORR showed that excluding the ASCENT study markedly reduced heterogeneity (*I^2^* = 39%, *Q*-test *P* = 0.20), with a pooled RR of 1.22 (95% CI 0.96–1.53, *P* = 0.10) that remained nonsignificant. Exclusion of any other single study did not substantially reduce heterogeneity (*I^2^* > 90% in all cases), and results remained nonsignificant (Supplementary Figure 2), suggesting that the ASCENT study may may have been the primary source of the overall high heterogeneity. Sensitivity analyses for PR yielded results consistent with those for OR (Supplementary Figure 3).

### 3.5 Safety outcomes

From a comprehensive safety evaluation pooling data from 966 patients on SG, the any-grade AEs with the highest reported incidence were neutropenia (679/966), followed by nausea (557/966), diarrhea (537/966), alopecia (496/966), anemia (404/966), and fatigue (402/966).

Among patients with mBC, SG did not confer a significantly different risk of anemia (RR 1.17, 95% CI 0.87–1.57; *P* = 0.30) compared with chemotherapeutic agents. However, SG treatment resulted in a significantly higher incidence of some specific AEs, including neutropenia, nausea, diarrhea, alopecia, and fatigue (all *P* < 0.05; see Figure 9 for detailed RR values and 95% CIs). Frequent grade ≥3 AEs in the SG group were neutropenia (*n* = 500), leukopenia (*n* = 138), diarrhea (*n* = 86), and anemia (*n* = 79). Importantly, SG significantly increased the risk of grade ≥3 neutropenia (RR 1.24, 95% CI 1.06–1.46; *P* = 0.009) and grade ≥3 diarrhea (RR 8.46, 95% CI 1.48–48.51; *P* = 0.02) compared with chemotherapy (Figure 10).

**Figure 9:**
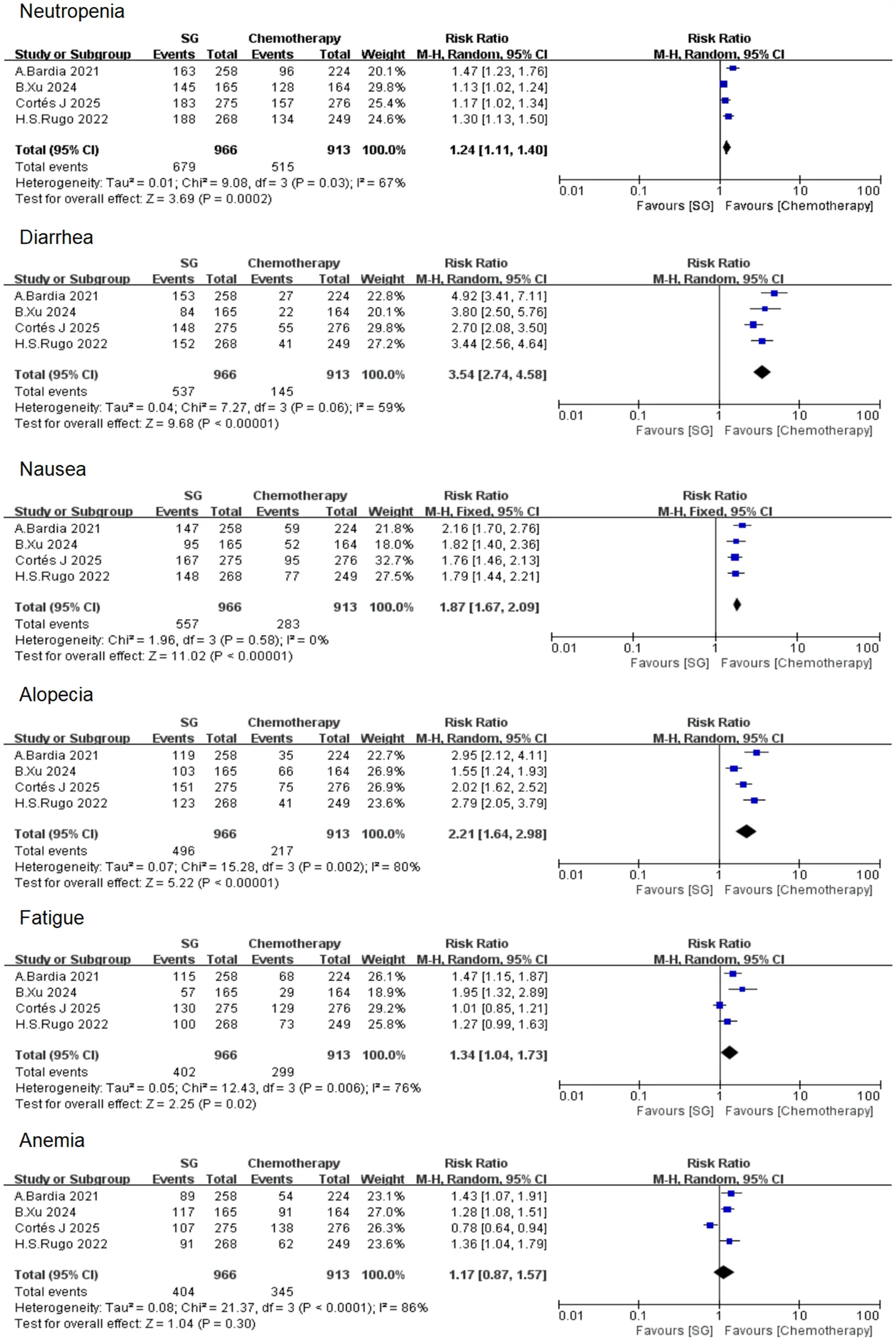
Forest plot of the risk ratios(RR) for AEs of any grade.

**Figure 10:**
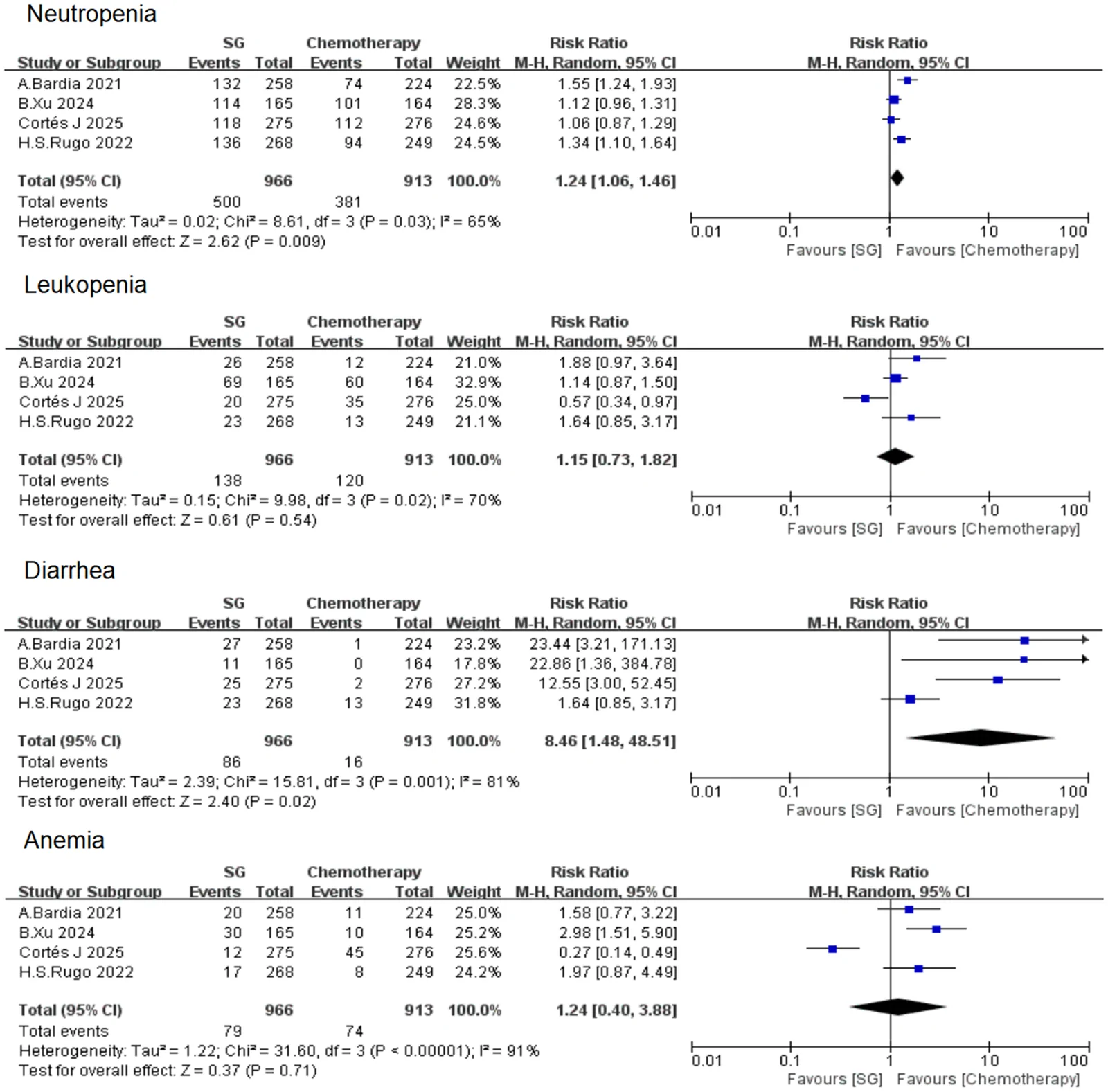
Forest plot of the risk ratios(RR) for grade ≥ 3AEs.

### 3.6 Analysis of Trop-2 expression: exploratory findings

Both the TROPiCS-02 and EVER-132-002 trials determined tumor Trop-2 expression by immunohistochemical staining, and membranous expression was measured by the H-score (histochemical score; range 0–300). SG showed a consistent survival benefit over chemotherapy across H-score subgroups (<100 and ≥100) in exploratory efficacy evaluations. In the EVER-132-002 trial, the PFS HR for SG versus chemotherapy was 0.91 (95% CI 0.65–1.28) in the Trop-2 H-score < 100 subgroup and 0.44 (95% CI 0.29–0.68) in the H-score ≥ 100 subgroup. For OS, the corresponding HRs were 0.72 (95% CI 0.47–1.10) and 0.61 (95% CI 0.37–1.01), respectively (23). The TROPiCS-02 trial similarly demonstrated that SG conferred an OS benefit across different subgroups. Furthermore, the supplementary efficacy evaluation in patients with H-score ≤ 10 provided further evidence of SG benefit; the median OS of the SG treatment group was 17.6 months, which was superior to the 12.3 months observed in the chemotherapy group (22).

Based on the above exploratory analyses, the therapeutic efficacy of SG across varying Trop-2 expression levels was preliminarily demonstrated. The key factors underlying this efficacy likely include the high affinity of SG for Trop-2 and its inherent bystander anti-tumor effect (25). However, because no validated immunohistochemistry scoring algorithm for Trop-2 has been established in breast cancer, and the sample sizes of these subgroups were relatively small, the analyses lacked sufficient statistical power to detect differences between subgroups, thereby limiting more definitive analysis and interpretation of the results.

### 3.7 Health-related quality of life

Both the oncological disease itself and treatment-related side effects from previous or ongoing lines of therapy can adversely affect functional well-being and overall health-related quality of life (HRQoL) in patients with mBC (26).

Three studies (21–23) reported patient-reported HRQoL using the EORTC QLQ-C30 at the beginning of each treatment cycle (Day 1). Overall, SG was associated with better HRQoL in patients with mBC, with improvements in global health status and physical functioning, as well as delayed deterioration. In ASCENT, time to first clinically meaningful deterioration in physical functioning, role functioning, fatigue, and pain was significantly longer with SG than with chemotherapy (all *P* < 0.05). TROPiCS-02 showed greater benefits with SG in physical functioning and dyspnea relief, as well as delayed deterioration in physical functioning, fatigue, and global health status/quality of life. EVER-132-002 yielded similar findings, with additional differences in pain. Patients in the SG group exhibited poorer outcomes in terms of gastrointestinal symptoms (nausea and diarrhea); however, these are known safety characteristics of SG and are manageable in accordance with current guideline recommendations (27, 28).

Because the present study included a limited number of trials and some reported results were not fully consistent, only descriptive summaries are provided. When sufficient data from additional studies become available in the future, more detailed quantitative pooled analyses of individual domains and items can be performed, thereby providing more robust scientific evidence regarding the value of SG as a treatment option for mBC.

## 4 Discussion

For women with mBC, which is characterized by rapid progression and pronounced resistance to conventional therapies, the disease poses a substantial risk to overall health and HRQoL. Chemotherapy, although still foundational, frequently yields only temporary responses and is limited by dose-cumulative adverse effects. ADCs have thus emerged to transform the therapeutic landscape (29). SG represents one of the leading ADCs in the clinical setting. It is built on the humanized monoclonal antibody hRS7 (IgG1*κ*), which targets Trop-2, a protein that is broadly expressed in tumor tissues and markedly overexpressed in up to 78% of TNBC (30). The cleavable CL2A linker connects SN-38 to the antibody at a high average DAR of about 7.6, a feature that markedly improves cytotoxic payload delivery to tumor cells (13). Being the first ADC against Trop-2 to gain approval for mBC, SG has become a model for precision-targeted cytotoxic therapy.

The ASCENT trial for metastatic TNBC demonstrated that, compared with chemotherapy, SG nearly tripled the median PFS and almost doubled the median OS (21). Subsequently, the TROPiCS-02 trial, which expanded the study population to include patients with HR+/HER2- mBC, further confirmed the clinical efficacy of SG in this breast cancer subtype (22). For the management of pretreated advanced or metastatic TNBC and HR+/HER2- breast cancer, SG was granted FDA approval based on these results (31). This meta-analysis of four RCTs (ASCENT, TROPiCS-02, EVER-132-002, and ASCENT-03) pooled survival and tumor response data from 1,900 patients with mBC and suggests potential clinically meaningful efficacy of SG treatment.

Compared with chemotherapy, SG was associated with a 42% lower risk of disease progression in patients with mBC, suggesting a potential PFS benefit. The four RCTs differed in baseline treatment history: the ASCENT trial enrolled heavily pretreated patients with metastatic TNBC, TROPiCS-02 and EVER-132-002 enrolled patients with HR+/HER2− disease, and ASCENT-03 enrolled patients with metastatic TNBC who had received no prior systemic therapy for advanced/metastatic disease. Sensitivity analysis showed that the PFS benefit remained statistically significant after excluding any single study. Considering similar treatment settings, the pooled analysis of the remaining three trials after excluding ASCENT-03 also suggested a treatment benefit in heavily pretreated mBC (HR = 0.57; 95% CI 0.41–0.78; *Z* = 3.54, *P* = 0.004). In exploratory subgroup analyses by tumor subtype, SG showed a treatment benefit in both TBBC and HR+/HER2− subgroups, with no statistically significant interaction between subgroups. Combined with the sensitivity analyses, the PFS benefit of SG in mBC appears reasonably robust. However, given the exploratory nature and limited statistical power of these subgroup analyses, this finding requires further validation and should not be equated with evidence from larger studies.

Because the ASCENT-03 trial's OS data were immature (37% maturity; SG 21.5 months [95% CI 17.7-NR] vs. chemotherapy 20.2 months [95% CI 18.2-NR]) (24), they were not incorporated into the current OS meta-analysis. Pooled analysis of three studies suggested a trend toward OS benefit with SG, corresponding to a risk of death 62% that of chemotherapy (HR 0.62). However, given the very limited number of included studies and the substantial heterogeneity, further sensitivity analyses indicated that the robustness of the pooled OS result was suboptimal. In particular, excluding the EVER-132-002 trial shifted the confidence interval upper limit to slightly above 1, placing the result at the borderline of statistical significance. Therefore, the OS benefit of SG should be interpreted cautiously, and future incorporation of more complete follow-up data is warranted to enhance the robustness and clinical applicability of the findings.

Pooled analysis of tumor response showed a trend toward higher ORR and PR rates with SG versus chemotherapy, but neither difference was statistically significant; the statistically significant difference in CR also requires cautious interpretation. The included trials varied in region, tumor subtype, and prior therapy, and this heterogeneity may account for the nonsignificant ORR and PR results. Moreover, CR is less common than PR in clinical practice, and in the CR forest plot, the ASCENT-03 trial carried a disproportionately high weight. Thus, despite statistical significance, the limited number of events and unbalanced weighting limit the clinical generalizability of the CR result, which should be regarded as exploratory. Larger, more homogeneous studies are needed for confirmation.

Given that ADCs possess a dual targeting mechanism that combines antigen specificity with potent cytotoxic effects, safety remains a central concern (32). The pooled analysis of 966 patients treated with SG in this meta-analysis revealed that the most frequently reported AEs included neutropenia, which occurred in approximately 70% of patients, followed by gastrointestinal toxicities such as nausea (58%) and diarrhea (56%). Among grade ≥3 AEs, SG was associated with an increased risk of grade ≥3 neutropenia and grade ≥3 diarrhea. However, the diarrhea result (RR 8.46,95% CI 1.48–48.51；*P* = 0.02) was imprecise owing to a wide confidence interval and substantial heterogeneity (*I^2^* = 81%, *Q* statistic *P* = 0.001). Therefore, we further evaluated absolute risk differences to supplement the clinical relevance. The risk difference (RD) model showed no significant heterogeneity among studies (I^2^ = 46%, *Q* statistic *P* = 0.13), with a fixed-effect pooled RD of 0.07 (95% CI 0.05–0.09; *P* < 0.01) (Figure 11), indicating an approximately 7% higher absolute risk of grade ≥3 diarrhea with SG than with chemotherapy. This estimate may serve as a more appropriate clinical reference.

**Figure 11:**
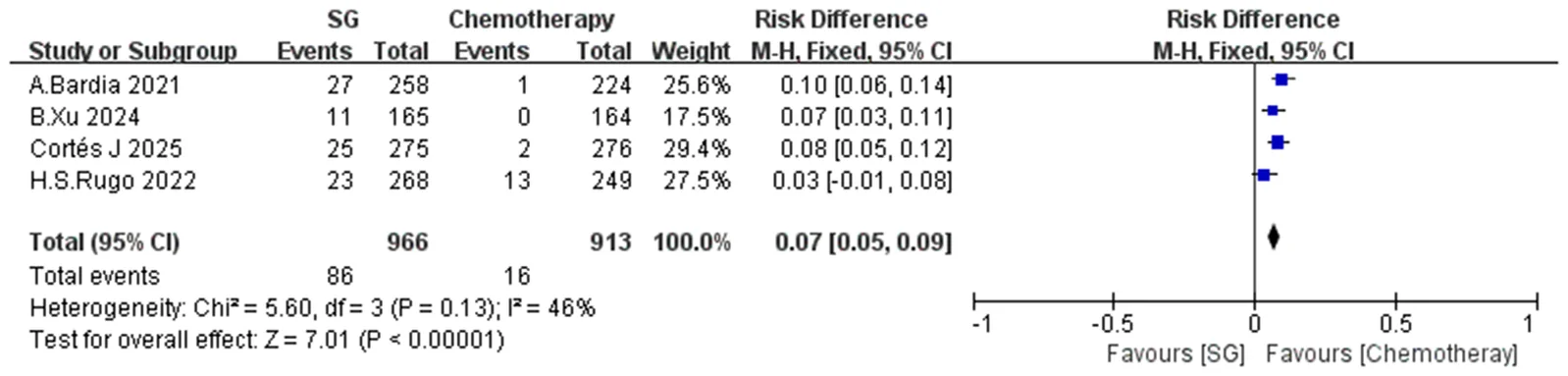
Forest plot of the absolute risk for diarrhea of grade ≥ 3.tables.

The theoretical basis for explaining the toxic effect of SG is related to its mechanism of action (33). Recognition of Trop-2 on benign tissues by SG can lead to on-target, off-tumor toxicity characterized by localized reactions. In parallel, premature systemic dissemination of SN-38, driven by linker cleavage or catabolic breakdown during the internalization process, accounts for off-target, off-tumor toxicity, presenting as gastrointestinal symptoms and hematologic adverse events (34). SN-38 blocks topoisomerase I, thereby inducing DNA double-strand breaks that persist and drive cell cycle arrest followed by apoptosis (35). This phenomenon is more commonly observed in rapidly proliferating cells, such as hematopoietic stem cells. Consequently, the most prevalent toxicities in patients administered SG were hematologic toxicities, predominantly neutropenia; the predominant grade ≥3 AEs were neutropenia (51.8%) and leukopenia (14%). The key gastrointestinal AEs associated with SG include nausea and diarrhea. Off-target, off-tumor toxicity is considered the predominant mechanism underlying diarrhea (32).

Moreover, the primary metabolic pathway of SG involves uridine diphosphate glucuronosyltransferase 1A1 (UGT1A1). The UGT1A1 enzyme converts SN-38 into its metabolite via a glucuronidation reaction, thereby facilitating drug excretion (36). Evidence indicates that UGT1A1 polymorphisms may be associated with adverse events during SG treatment (37). With ongoing breakthroughs in predictive biomarkers and advances in monitoring technologies, further optimization of the safety management and dosing strategies for SG therapy is anticipated (38).

Although SG is associated with certain toxicities, the treatment discontinuation rates reported across the studies were relatively low, and most fatal events were unrelated to treatment (17). The implementation of AEs management strategies, such as the administration of granulocyte colony-stimulating factor and antiemetic agents, can effectively alleviate clinical symptoms, rendering SG clinically acceptable for the majority of patients.

This meta-analysis has several limitations. The number of included trials was very limited, data for some endpoints were immature, and the small number of studies resulted in insufficient statistical power for subgroup analyses and precluded formal assessment of publication bias. Only English-language literature was included, which may have introduced selection bias, although no eligible non-English-language studies were identified during the search. In addition, substantial clinical heterogeneity across studies, including differences in molecular subtypes and prior treatment baselines, remained unavoidable, and the impact of nonclinical factors such as ethnic differences, healthcare policies, infrastructure, and cultural differences on study outcomes is difficult to quantify. These limitations may restrict the interpretation and generalizability of the efficacy findings to the overall mBC population. Furthermore, this study included only clinical trials of SG monotherapy. The clinical efficacy of SG in the early-stage disease setting has only recently begun to be investigated, and multiple clinical trials are underway to evaluate its therapeutic potential in the neoadjuvant setting and in combination regimens with immunotherapy (39, 40). We have endeavored to present an objective account of the current evidence and to focus on exploring the potential value of this therapeutic regimen. As the evidence matures in the future, more comprehensive and multidimensional analyses should be conducted to further delineate the therapeutic role of SG in breast cancer.

## 5 Conclusion

The introduction of Trop-2-directed ADCs has enriched the treatment armamentarium for mBC. Based on the currently available limited evidence, SG may provide clinical benefit and favorable effects on HRQoL in mBC; however, its more definitive clinical advantages and the magnitude of benefit still require further validation. In addition, SG is associated with toxicities including neutropenia and diarrhea, warranting appropriate prophylactic measures in clinical practice. Future RCTs and biomarker-driven studies will be critical to defining the position of SG within the therapeutic landscape for mBC.

## Data Availability

The original contributions presented in the study are included in the article/Supplementary Material, further inquiries can be directed to the corresponding author.

## Supplementary material

SUPPLEMENTARY FIGURE 1 Sensitivity analysis of PFS using a fixed-effect model.

SUPPLEMENTARY FIGURE 2 Sensitivity analysis of ORR.

SUPPLEMENTARY FIGURE 3 Sensitivity analysis of PR.
